# Tetrasodium EDTA Is Effective at Eradicating Biofilms Formed by Clinically Relevant Microorganisms from Patients’ Central Venous Catheters

**DOI:** 10.1128/mSphere.00525-18

**Published:** 2018-11-28

**Authors:** Fangning Liu, Satyender Hansra, Gordon Crockford, Wolfgang Köster, Brenda J. Allan, Joseph M. Blondeau, Chantal Lainesse, Aaron P. White

**Affiliations:** a Vaccine and Infectious Disease Organization-International Vaccine Centre, Saskatoon, Saskatchewan, Canada; b Department of Veterinary Microbiology, University of Saskatchewan, Saskatoon, Saskatchewan, Canada; c Division of Clinical Microbiology, Royal University Hospital and Saskatoon Health Region, Saskatoon, Saskatchewan, Canada; d Department of Pathology and Ophthalmology, University of Saskatchewan, Saskatoon, Saskatchewan, Canada; e Department of Microbiology and Immunology, University of Saskatchewan, Saskatoon, Saskatchewan, Canada; f SterileCare, Inc., Markham, Ontario, Canada; Escola Paulista de Medicina/Universidade Federal de São Paulo

**Keywords:** EDTA, fungi, Gram-negative, Gram-positive, antibiotic resistance, antimicrobial, biofilms, catheters, central venous access devices, minimum inhibitory concentration

## Abstract

Central venous access devices (CVADs) are an essential component of modern health care. However, their prolonged use commonly results in microbial colonization, which carries the potential risk of hospital-acquired bloodstream infections. These infections complicate the treatment of already sick individuals and cost the existing health care systems around the world millions of dollars. The microbes that colonize CVADs typically form multicellular biofilms that are difficult to dislodge and are resistant to antimicrobial treatments. Clinicians are searching for better ways to extend the working life span of implanted CVADs, by preventing colonization and reducing the risk of bloodstream infections. In this study, we analyzed 210 bacterial and fungal isolates from colonized CVADs or human bloodstream infections from two hospitals geographically separated in the east and west of Canada and screened the isolates for biofilm formation *in vitro*. Twenty isolates, representing 12 common, biofilm-forming species, were exposed to 4% tetrasodium EDTA, an antimicrobial lock solution that was recently approved in Canada for use as a medical device. The EDTA solution was effective at eradicating surface-attached biofilms from each microbial species, indicating that it could likely be used to prevent biofilm growth within CVADs and to eliminate established biofilms. This new lock solution fits with antibiotic stewardship programs worldwide by sparing the use of important antibiotic agents, targeting prevention rather than the expensive treatment of hospital-acquired infections.

**IMPORTANCE** The colonization of catheters by microorganisms often precludes their long-term use, which can be a problem for human patients that have few body sites available for new catheters. The colonizing organisms often form biofilms, and increasingly these organisms are resistant to multiple antibiotics, making them difficult to treat. In this article, we have taken microorganisms that are associated with biofilm formation in catheters from two Canadian hospitals and tested them with tetrasodium EDTA, a new antimicrobial catheter lock solution. Tetrasodium EDTA was effective at eliminating Gram-positive, Gram-negative, and fungal species and represents a promising alternative to antibiotic treatment with less chance of the organisms developing resistance. We expect that our results will be of interest to researchers and clinicians and will lead to improved patient care.

## Abstract (precis)

The colonization of catheters by microorganisms often precludes their long-term use, which can be a problem for human patients that have few body sites available for new catheters. The colonizing organisms often form biofilms, and increasingly these organisms are resistant to multiple antibiotics, making them difficult to treat. In this article, we have taken microorganisms that are associated with biofilm formation in catheters from two Canadian hospitals and tested them with tetrasodium EDTA, a new antimicrobial catheter lock solution. Tetrasodium EDTA was effective at eliminating Gram-positive, Gram-negative, and fungal species and represents a promising alternative to antibiotic treatment with less chance of the organisms developing resistance. We expect that our results will be of interest to researchers and clinicians and will lead to improved patient care.

## INTRODUCTION

Hospital-acquired infections (HAIs) are often caused by multidrug-resistant bacteria such as methicillin-resistant Staphylococcus aureus (MRSA) and Pseudomonas (1). HAIs are typically difficult to treat and add complications, such as prolonged hospital stays and death, to an already sick patient (2–4). Incidences of HAI are thought to be underreported due to the use of inefficient medical tracking systems and fear of potential organizational repercussions. One known risk for HAI is the use of central venous access devices (CVADs) (5). At least 70% of all nosocomial bloodstream infections occur in patients who have CVADs (6, 7). This includes patients in intensive care, patients with cancer, and patients who are in need of hemodialysis or total parenteral nutrition (TPN) (8–10).

There is evidence that colonized CVADs can be important carriers of MRSA and other multidrug-resistant bacteria in communities and hospitals worldwide (2, 6). Three processes that can cause CVAD-related complications are clot formation, microbial colonization, and biofilm formation, also known as the TripleThreat (6, 7, 11). It is no longer sufficient to simply prevent clots without providing protection against microorganisms and their associated biofilms (12, 13). Biofilms have a specialized physiology where cells aggregate together and become encased in a self-produced polysaccharide and protein matrix that protects the cells from harsh environmental elements (14–16). Harsh conditions for microorganisms within a CVAD would include mechanical flushing and the use of antibiotic agents (6). Each time the CVAD is used, there is potential for resistant microorganisms sloughing off from a biofilm and entering the patient’s bloodstream (6, 11, 17, 18).

Infection control practitioners and catheter management teams worldwide have been aggressively searching for antimicrobial lock solutions to disinfect and keep the inside of the catheters free of microorganisms (7, 19). There is currently no approved lock solution that can effectively protect CVADs against the TripleThreat (12). The current standard of care in many parts of the United States and other areas of the world is heparin or saline (12, 20). Heparin is a well-characterized anticoagulant but has known safety risks, including causing low platelet counts and/or heparin-induced thrombocytopenia; there is also evidence that heparin can stimulate biofilm growth (21). Recently, a 4% tetrasodium ethylenediamine tetraacetic acid (EDTA) lock solution was approved by Health Canada to maintain patency and decrease the risk of bacterial colonization and biofilm formation within CVADs. EDTA has been used for decades as a powerful anticoagulant preventing clot formation *in vitro* (22, 23) and is still being used in this capacity today (24). However, the form of EDTA most often used is the disodium salt, which has reduced antimicrobial properties (25). In contrast, the tetrasodium salt of EDTA has shown the ability to disrupt *in vivo-* and *ex vivo-*generated biofilms (26, 27) and in a randomized, controlled clinical trial showed significant improvement compared to heparin (28).

The most important quality measure for a proposed CVAD treatment is the rate of central-line-associated bloodstream infections (CLABSIs). However, determination of the CLABSI rate suffers from subjectivity and variability, because there is not always a direct link between colonization and known CLABSI events. Not all catheters colonized with bacteria will result in CLABSI, but there is an increase in CLABSI risk when bacteria are detected within catheters (29–31). Rijinders and colleagues established that there is a linear correlation between catheter tip culture (CTC) and CLABSI and that CTC can be used as a surrogate endpoint for CLABSI when catheters are removed (32). It is estimated that a positive bacterial CTC reading has a 20% chance of resulting in a CLASBI event (33–35). Thus, although it is not a perfect measure, investigating the prevention of CTC can be useful for reducing CLABSI prevalence.

The goals in our study were 2-fold: (i) to identify microorganisms isolated by CTC or from human bloodstream infections and test their biofilm-forming ability and (ii) to evaluate the efficacy of tetrasodium EDTA at eliminating biofilms, toward its use as an antimicrobial lock solution.

## RESULTS

### Isolation of microorganisms from patients’ central venous catheters.

As part of a collaborative study with clinicians and nurses at the Southlake Regional Health Center (SRHC) in Ontario (ON), catheters were collected from patients over an 8-month period and cultured for microorganisms using a traditional roll-plate technique coupled with sonication (33–35). A total of 168 isolates were cultured from a total of 305 catheters. This equates to an ∼50% isolation and/or colonization rate, since very few catheters had more than one species cultured (data not shown). The isolates obtained belonged to 33 different species, including Gram-negative and Gram-positive bacteria and fungi (Table 1). The most predominant single species was Staphylococcus epidermidis, with 66 isolates, which represented 39% of the total. All 11 isolates of S. aureus were independently identified to the species level by matrix-assisted laser desorption ionization–time of flight mass spectrometry (MALDI-TOF MS) and confirmed prior to testing for antibiotic resistance. One isolate was identified as MRSA.

**TABLE 1:** Bacterial and fungal isolates cultured from central venous access devices or human blood samples

Organism type	No. of isolates	% of total
Ontario (CVADs)^*a*^		
Gram-positive bacteria	120	71.4
Staphylococcus epidermidis	66	39.3
Staphylococcus aureus	11^*b*^	6.5
Other Staphylococcus spp.^*c*^	20	11.9
Bacillus spp.^*d*^	9	5.4
Corynebacterium spp.^*e*^	5	3.0
Enterococcus faecalis	2	1.2
Other Gram-positive species^*f*^	7	4.2
Gram-negative bacteria	34	20.2
Ralstonia insidiosa	6	3.6
Stenotrophomonas maltophilia	5	3.0
Enterobacter agglomerans	4	2.4
Proteus mirabilis	3	1.8
Escherichia coli	2	1.2
Other Gram-negative spp.^*g*^	14	8.3
Fungi	14	8.3
Candida albicans	10	6.0
Candida glabrata	4	2.4
Ontario total	168	
Saskatchewan (blood samples)^*h*^		
Gram-positive bacteria	15	NA^*j*^
Staphylococcus epidermidis	12	
MRSA	3	
VRE^*i*^	3	
Gram-negative bacteria	12	NA
Pseudomonas aeruginosa	3	
Klebsiella pneumoniae	3	
Serratia marcescens	3	
Escherichia coli	3	
Fungi	15	NA
Candida albicans	12	
Candida glabrata	3	
Saskatchewan total	42	

a Isolates from Ontario were cultured from 305 catheter tips removed from patients at Southlake Regional Health Centre.

b One S. aureus isolate was classified as methicillin resistant (MRSA).

c Additional Staphylococcus species included S. lugdunensis (7 isolates), S. hominis (6 isolates), S. simulans (2 isolates), S. capitis (2 isolates), and undetermined (3 isolates).

d Bacillus species included B. licheniformis (2 isolates), B. megaterium (2 isolates), B. simplex (1 isolate), B. cereus group (1 isolate), and undetermined (3 isolates).

e Corynebacterium species included C. tuberculostearicum (2 isolates) and undetermined, not C. jeikeium (3 isolates).

f Additional Gram-positive species included Streptococcus mitis (1 isolate), Nocardia spp. (1 isolate), Paenibacillus spp. (1 isolate), and undetermined (4 isolates).

g Additional Gram-negative species included Comamonas testosteroni (3 isolates), Sphingomonas paucimobilis (3 isolates), Brevundimonas spp. (2 isolates), Pseudomonas orizyhabitans (2 isolates), Ralstonia pickettii (1 isolate), Roseomonas gilardii (1 isolate), Rothia spp. (1 isolate), and undetermined (1 isolate).

h Isolates from Saskatchewan were cultured from patient blood samples from Royal University Hospital in Saskatoon.

i VRE, vancomycin-resistant Enterococcus faecalis.

j NA, not applicable.

In addition to the ON isolates, isolates corresponding to nine different bacterial and fungal species were obtained from patients at the Royal University Hospital, Saskatoon, Saskatchewan (SK) (Table 1). The nine species represented common, biofilm-forming species that have been associated with CVAD colonization (36) and included resistant pathogens such as MRSA and vancomycin-resistant enterococci (VRE). The isolates came from patient specimens submitted for culture and susceptibility testing, but the numbers associated with catheter use are unknown.

### Screening isolates for biofilm formation.

**FIG 1:**
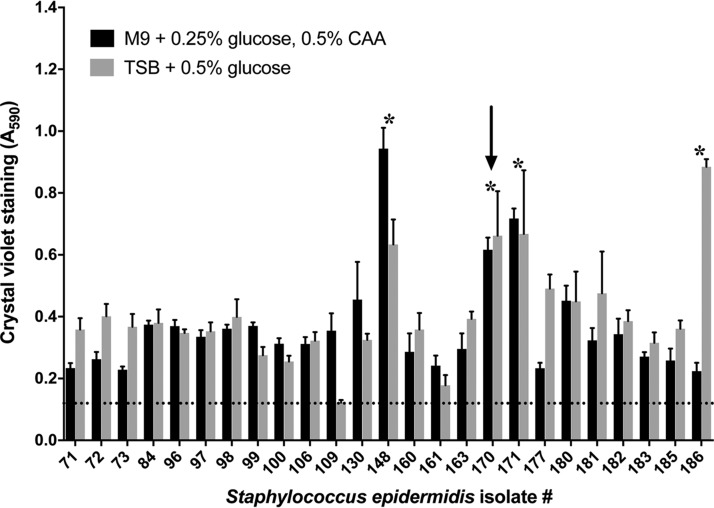
Biofilm screening of S. epidermidis isolates. Twenty-five S. epidermidis isolates originating from central venous access devices were inoculated into 96-well plates and grown for 24 h at 37°C in biofilm media: M9, M9 minimal media; CAA, Casamino Acids; TSB, tryptic soy broth. Biofilm cell mass in each well was quantitated by crystal violent staining and measuring the absorbance of the resulting solution at 590 nm (*A*_590_). Bars represent the average values and error bars the standard deviations from 6 biological replicates. The dashed horizontal line represents the average *A*_590_ value from uninoculated control wells. Stars denote isolates that were judged to have robust biofilm formation. Isolate 170 (arrow) was chosen for subsequent testing.

The 66 S. epidermidis isolates from Ontario were screened for biofilm formation in a 96-well plate format by growth in multiple growth media (see Table S1 in the supplemental material). Representative biofilm data are shown for a subset of 25 S. epidermidis isolates tested in two established biofilm media (Fig. 1). In this assay, four isolates formed robust biofilms with crystal violet (CV) staining at ≥2 to 4 times or >4 times background (37), and one isolate was chosen for further testing (Fig. 1, arrow). In total, 9 (13.4%) of 66 S. epidermidis isolates were identified as moderate to strong biofilm formers.

10.1128/mSphere.00525-18.3 TABLE S1 Biofilm screening conditions tested on SK and ON microbial isolates. Download Table S1, PDF file, 0.1 MB. Copyright © 2018 Liu et al. 2018 Liu et al. This content is distributed under the terms of the Creative Commons Attribution 4.0 International license.

Similar biofilm screening was performed for each bacterial and fungal species in the ON collection with at least two independent isolates and for all nine bacterial and fungal species originating from the SK collection. The goal was to identify one or more isolates from each species that formed robust and reproducible biofilms that could be further tested. For each species, we identified at least one strong biofilm-forming isolate, with the exception of Ralstonia insidiosa (data not shown). The antimicrobial resistance patterns were determined for each of these biofilm-forming isolates (see Table S2 in the supplemental material).

10.1128/mSphere.00525-18.4 TABLE S2 Antibiotic susceptibility profiles for biofilm-forming microbes isolated from Ontario and Saskatchewan hospitals. Download Table S2, PDF file, 0.08 MB. Copyright © 2018 Liu et al. 2018 Liu et al. This content is distributed under the terms of the Creative Commons Attribution 4.0 International license.

### Determination of MIC, MBC, and MBEC values for tetrasodium EDTA.

**FIG 2:**
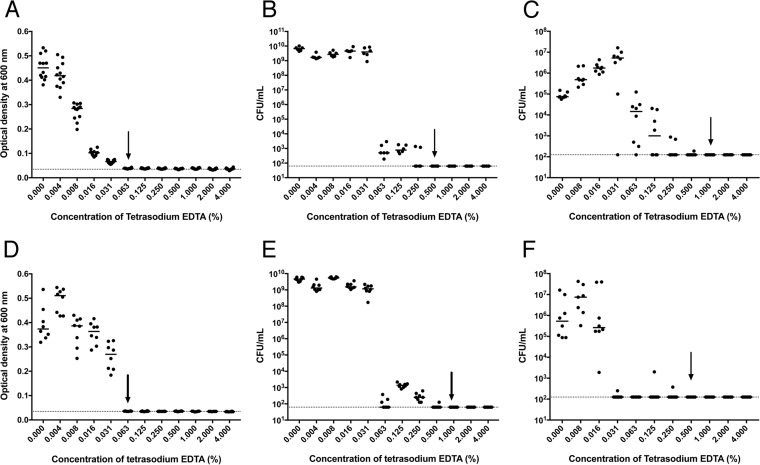
Determination of MIC, MBC, and MBEC values of tetrasodium EDTA against Staphylococcus isolates cultured from central venous access devices. Individual isolates of S. epidermidis (A, B, and C) and methicillin-resistant S. aureus (D, E, and F) were tested in MIC (A and D), MBC (B and E), and MBEC (C and F) assays. Horizontal bars represent the mean OD_600_ or viable bacterial cell (CFU/ml) values after cultures were exposed to increasing amounts of tetrasodium EDTA. Arrows represent MIC, MBC, and MBEC values. The dashed horizontal lines on each graph represent the background OD_600_ values in uninoculated control wells (A and D) or the CFU limit of detection (B, C, E, and F). Three biological replicate cultures were tested in duplicate or triplicate for each type of assay; each dot represents one replicate.

For the strong biofilm-forming isolates identified, we tested the MIC and minimum bactericidal concentration (MBC) for tetrasodium EDTA, when the isolates were grown as single cells in liquid culture. Representative data are shown for ON S. epidermidis and MRSA isolates (Fig. 2). Both isolates had no visible signs of growth in the presence of 0.063% tetrasodium EDTA as measured by optical density (OD) (Fig. 2A and D), whereas complete killing was achieved at 0.5% and 1.0% tetrasodium EDTA, respectively (Fig. 2B and E). To test the resistance of isolates when grown as biofilms, minimum biofilm eradication concentration (MBEC) assays were performed after growth of organisms on the surface of polystyrene pegs. MBEC values of 1.0% and 0.5% (Fig. 2C and F), which were similar to the MBC values, were obtained for both strains.

The MIC, MBC, and MBEC values for tetrasodium EDTA were determined for one or more isolates from each selected species originating from the ON and SK hospitals (Table 2). In general, MBC values were higher than the corresponding MIC tests, indicating that tetrasodium EDTA was not bactericidal at lower concentrations. Gram-negative bacteria and Candida isolates were more resistant, with MIC and MBC values that were, on average, 2 to 3 dilutions higher than those of the Gram-positive bacterial isolates. The concentration of tetrasodium EDTA required for killing biofilm cells was 4% for 8 of 20 tested isolates, whereas the remaining 12 isolates had MBEC values below 4% (Table 2).

**TABLE 2:** Effectiveness of tetrasodium EDTA at killing clinically relevant microorganisms grown as single cells or as biofilms

Organism type^*a*^	Result (%) by:			Biofilm killing^*b*^
	MIC	MBC	MBEC	
Gram-positive bacteria				
Staphylococcus epidermidis				
ON	0.063	0.5	1.0	4.2
SK	0.063	0.5	2.0	3.7
Staphylococcus aureus				
ON	0.063	1.0^*c*^	4.0	6.0
Methicillin resistant				
ON	0.063	1.0	0.5	4.6
SK	0.063	2.0^*c*^	4.0	4.4
Enterococcus faecalis				
ON	0.063	2.0	4.0	3.7
Vancomycin resistant (SK)	0.031	2.0	0.25	1.8

Gram-negative bacteria				
Escherichia coli				
ON	0.5	1.0	1.0	5.6
SK	0.125	0.25	2.0	4.4
Stenotrophomonas maltophilia (ON)	0.063	1.0	4.0	6.5
Pseudomonas aeruginosa (SK)	0.25	1.0	4.0	5.3
Enterobacter agglomerans (ON)	0.125	0.25	4.0	5.1
Serratia marcescens (SK)	1.0	1.0	4.0	5.0
Proteus mirabilis (ON)	0.063	2.0	4.0	5.7
Klebsiella pneumoniae (SK)	1.0	1.0	2.0	4.6

Fungi				
Candida albicans				
ON	1.0	2.0	1.0	2.7
SK	1.0	1.0	1.0	1.7
Candida glabrata				
ON	0.25	2.0	1.0	1.9
SK	0.125	2.0	1.0	2.0

Control bacteria				
Salmonella enterica serovar Typhimurium	0.25	0.5	1.0	4.7

a Microorganisms were obtained from Southlake Regional Health Centre in Ontario (ON) and Royal University Hospital in Saskatchewan (SK).

b These numbers refer to the log_10_ reductions of differences between the mean starting number of cells in the biofilm and the mean number of remaining cells after treatment with tetrasodium EDTA for 24 h at the concentrations listed in the MBEC column.

c MBC was determined by confirming the lack of surviving cells through inoculation of the treated culture into fresh medium and growth for 24 h at 37°C.

The clinically accepted standard of killing of microorganisms by an antimicrobial agent is at least a 3-log reduction in cell numbers (38). To visualize this standardized level of killing, the MBEC data from each experiment were plotted as the log reduction in the number of colony-forming units (see Fig. S1 in the supplemental material). For 13 of 20 tested isolates, greater than 4-log killing was achieved (Table 2). For the remaining 7 isolates, the starting biofilm cell densities were not high enough to achieve a 4-log reduction. However, in each case, 4% tetrasodium EDTA was able to kill biofilm cells down to the limit of detection for the assay (Fig. S1).

10.1128/mSphere.00525-18.2 FIG S1 Eradication of bacterial and fungal biofilms upon exposure to tetrasodium EDTA. *In vitro* biofilms formed by Gram-positive bacteria (A), Gram-negative bacteria (B), fungal species (C), and control bacteria (D) on polystyrene pegs were exposed to tetrasodium EDTA (4.000, 2.000, 1.000, 0.500, 0.250, 0.125, 0.063, 0.031, 0.016, and 0.008%) and control medium for 24 h. Data are presented as the log reduction in CFU/ml values from the starting amount of biofilm and each treatment group; the solid horizontal line and shaded area below represent a 4-log reduction or 99.99% killing. Bars represent the average values (*n* = 6 or 8), and error bars represent 1 standard deviation. Values from each treatment group were compared to the medium control group using the uncorrected Dunn’s test of Kruskal-Wallis test, with statistical significance noted under the bar (*, *P < *0.01). Arrows denote the minimum biofilm eradication concentration (MBEC). Download FIG S1, PDF file, 0.6 MB. Copyright © 2018 Liu et al. 2018 Liu et al. This content is distributed under the terms of the Creative Commons Attribution 4.0 International license.

### Exposure time required to kill *in vitro*-formed biofilms.

**FIG 3:**
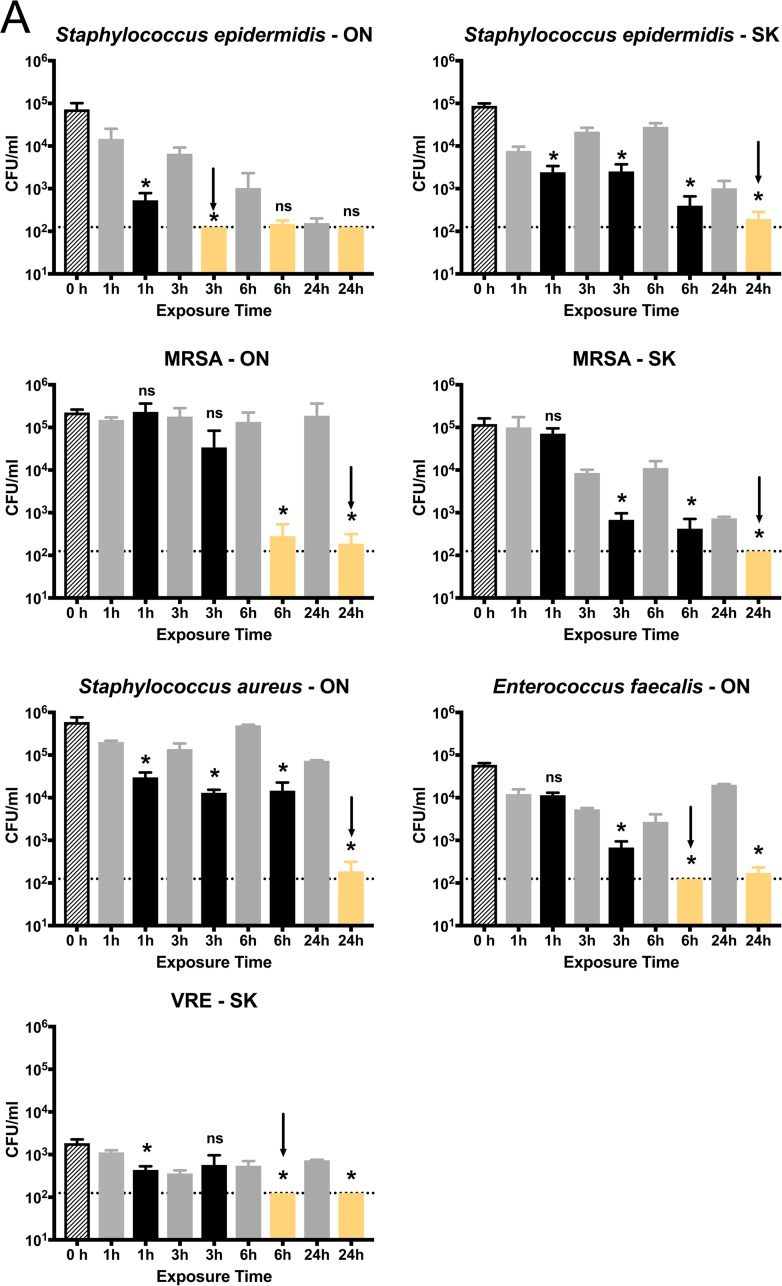

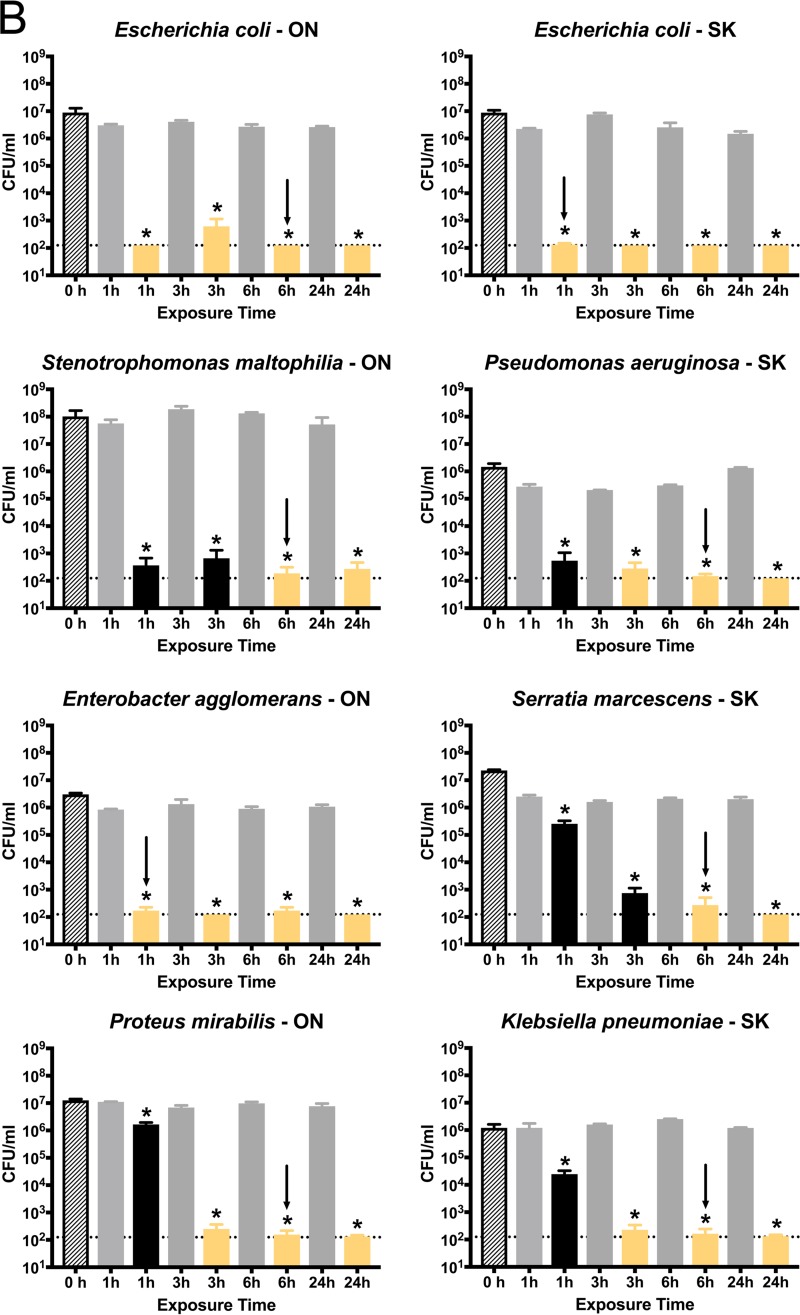

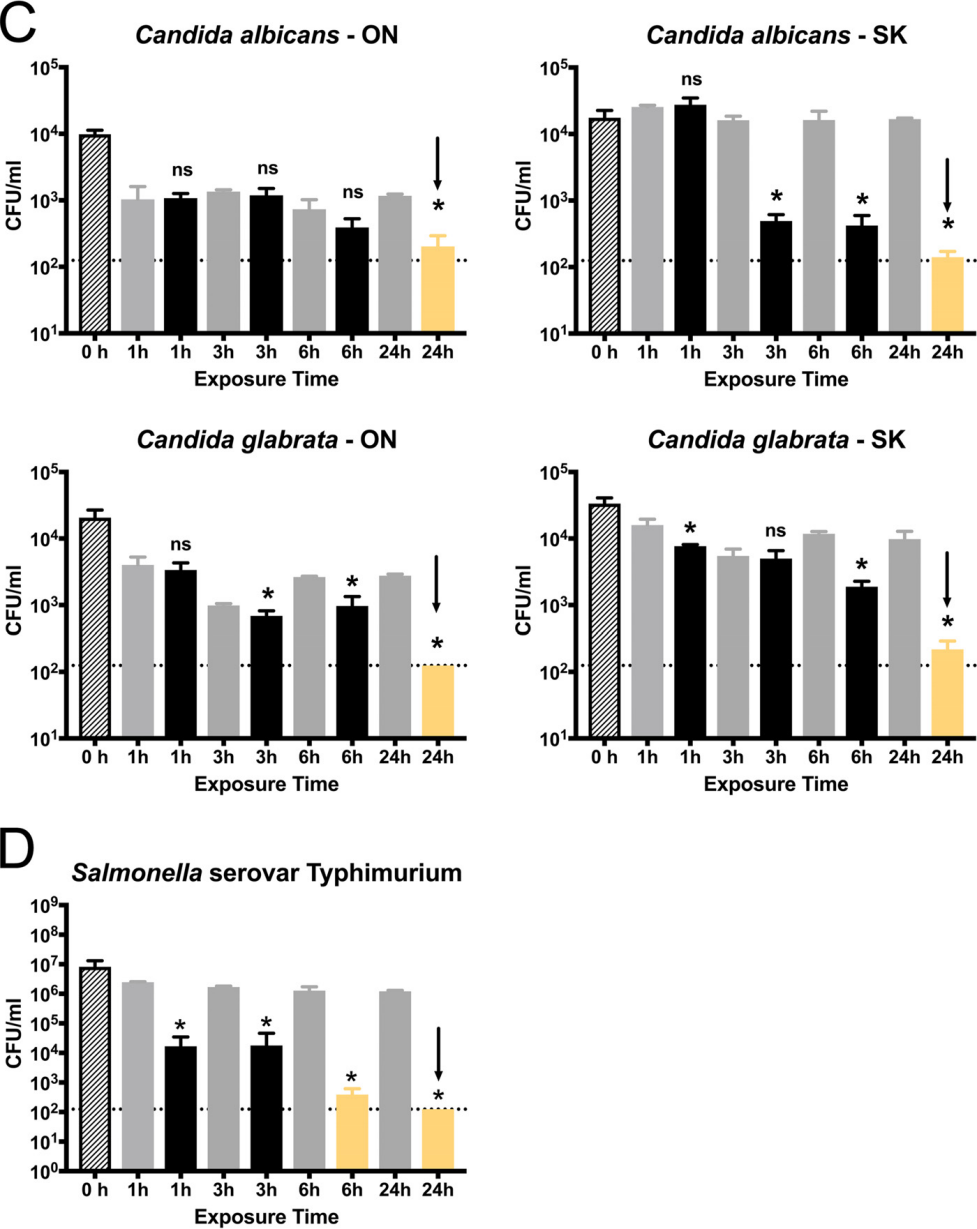
Minimum exposure time to kill bacterial/fungal biofilms with 4% tetrasodium EDTA. *In vitro* biofilms formed by Gram-positive bacteria (A), Gram-negative bacteria (B), fungal species (C), and control bacteria (D) were formed on polystyrene pegs prior to testing. For each graph, the hatched bar (0 h) shows the starting CFU/ml values measured from control pegs (*n* = 8). Formed biofilms were exposed to 4% tetrasodium EDTA (black bars) or water (gray bars) for the times shown; four biological replicates with four technical replicates (*n* = 16) were analyzed for treatment groups, along with four biological replicates with two technical replicates (*n* = 8) for water controls. Bars represent the average CFU/ml detected, and error bars represent the standard deviation. The time points where biofilms were killed near or at the limit of detection (dotted line [125 CFU/ml]) are highlighted in yellow. Values from each treatment group were compared to the corresponding water controls by unpaired *t* tests with Welch’s correction. Statistical significance is noted above each treatment bar: ns, not significant (*P > *0.05); *, *P < *0.05. Arrows denote the minimum exposure times required for complete eradication of the bacterial/fungal biofilms.

The exposure time for MBEC assays was set to 24 h to follow established guidelines (39). However, there are several clinical scenarios in which catheter lock solutions could be used for less than 24 h. To establish the minimum contact time necessary for complete biofilm killing to occur, we tested each selected biofilm-forming isolate by exposure to 4% tetrasodium EDTA for 1, 3, 6, or 24 h (Fig. 3). In these time-to-kill assays, tetrasodium EDTA was directly compared to a water control, which accounts for cells that may slough off the pegs in a passive manner compared to cells that are actively killed.

All Gram-positive isolates showed a statistically significant drop in CFU compared to controls after 6 h of exposure (Fig. 3A). The S. epidermidis isolate from Ontario was completely killed after 3 h, whereas the three S. aureus isolates required longer exposure times to drop CFU values near the limit of detection, and a full 24 h was required for complete killing. For Enterococcus faecalis, although biofilm formation for both ON and SK isolates was not as robust as that of the other Gram-positive species, complete killing was achieved within 6 h. The results with Gram-negative bacteria were quite different. All 8 isolates had killing at or near the limit of detection within 3 h of exposure, and none required a full 24 h of exposure (Fig. 3B). Six of eight Gram-negative isolates had complete killing achieved by 6 h. Finally, Candida species appeared to be the most resistant to tetrasodium EDTA, as each selected isolate required a full 24 h of exposure for complete killing to occur (Fig. 3C).

## DISCUSSION

There is currently a great need for the development of effective nonantibiotic antimicrobials that can be used in a clinical setting. Antibiotic resistance of microorganisms is a huge problem and is anticipated to increase in severity with time (40, 41). In this study, we tested the ability of 4% tetrasodium EDTA to kill clinically significant bacterial and fungal pathogens isolated from two Canadian hospitals. The tetrasodium EDTA solution was able to kill all microorganisms tested, at a concentration of 4% or less, and in less than 24 h of exposure. We also tested organisms when grown as biofilms, which represents a worst-case scenario for the colonization of catheters (6, 7) and contributes to numerous clinical diseases (42, 43). As anticipated, biofilms were the most difficult physiology to eradicate; however, clinically significant levels of killing were achieved (i.e., 4-log reduction in CFU or 99.99% killing) for 13 of 20 isolates tested. For the remaining seven isolates, the initial biofilm density was not as high, so even though cells were killed at or below detectable levels, 4-log killing could not be achieved. These results indicated that 4% tetrasodium EDTA was an effective antimicrobial agent against all tested Gram-positive and Gram-negative bacteria and fungi coming from patients.

Microbial colonization of CVADs is known to be a major contributing factor to CLABSI, HAI, and the spread of antibiotic resistance (6, 19, 44). In our study, the 54% rate of bacterial and fungal isolate identification from 305 catheters was similar to what has been reported before (33). The Maki roll technique, which has low sensitivity for isolation of intraluminal bacteria (34), was combined with sonication to maximize the chances of detecting bacterial colonization. Recently, culture-independent approaches have shown that both “symptomatic” and “nonsymptomatic” catheters contain diverse microorganisms (45–47), with “symptomatic” catheters colonized at higher levels and the most numerically dominant organisms often being the only ones cultured. This could explain the commonly held dogma that some catheters are colonized and others are not. In our study, the isolated organisms likely represent a mix of attached, surface- or lumen-associated cells (33, 34). We obtained diverse Gram-negative, Gram-positive, and fungal isolates, and S. epidermidis was the most predominant single species, which matches well with previous reports (36, 45, 47). However, if we had employed a culture-independent approach focused on sequencing, we anticipate that microorganisms would have been detected in nearly 100% of the catheters.

More research is needed to better understand what signals trigger biofilm development in the host and to learn how to extrapolate these signals to the *in vitro* environment. Although it is difficult to know the true prevalence of *in vivo* biofilm formation, recent studies using microscopic examination of the inside of catheters indicate that it could be near 100% (45–47). Despite this high *in vivo* prevalence, only 10 to 15% of isolates in our study were capable of forming robust biofilms *in vitro.* This was particularly surprising for S. epidermidis, which is the number 1 species associated with catheter biofilms *in vivo* in Canada (36) and in other areas in the world (48, 49). We hypothesize that the inability of most isolates to make biofilms *in vitro* is due to the difficulty in reproducing *in vivo* conditions. In light of recent sequencing results, it is possible that most or all catheter biofilms are multispecies, which could mean that microorganisms need to interact for efficient attachment to occur and to foster the development of a mature biofilm matrix (43, 44, 50, 51). Perhaps the stepwise addition of organisms is required, as has been observed for microbial biofilms formed on human teeth (52). There is also a strong possibility that microbial cells are in a different metabolic state *in vivo* due to the presence of blood and trace elements, as well as oxygen and nutrient gradients (53, 54). Many of these factors are difficult to control in the laboratory setting, but each could play a role in stimulating microbial attachment (55, 56). As we have done here, researchers often characterize biofilm-forming “type” strains and make the assumption that they are representative of the entire species. It should also be noted that quantitation of *in vitro* biofilms is inherently flawed, since crystal violet staining is an indirect measurement of biomass that suffers from a lack of reproducibility, and sonication only removes a proportion of bound cells from the polystyrene pegs of MBEC devices.

Treatment of monoculture biofilms with tetrasodium EDTA revealed different susceptibilities, depending on the class of organism. Gram-negative isolates were killed in the shortest exposure time but generally required higher concentrations of tetrasodium EDTA, presumably to overcome the stringent outer and inner membrane barriers (57). In contrast, Gram-positive isolates took longer to kill but were killed at lower concentrations. We are not sure why this difference was detected, but it could reflect a difference in biofilm architecture compared to Gram-negative organisms. Perhaps lack of an outer membrane in Gram-positive bacteria renders them susceptible to lower concentrations of EDTA. Candida species, which can exist in both cellular and hyphal forms (58–60), were the most difficult to treat, requiring the highest concentrations and exposure times. The fact that tetrasodium EDTA worked against all three classes of organisms confirms that it has wide-ranging antimicrobial effects. The majority of effects are assumed to be due to its chelation activity, including outer membrane damage and changes to chromosomal and RNA activity (61–64). With EDTA predicted to have more generalized mechanism of killing, we hypothesize that it would be difficult for microorganisms to develop resistance. EDTA can also increase the permeability of microbial membranes (62, 65), suggesting that it could be used synergistically with other antimicrobial compounds or with low levels of antibiotics that have fallen out of use. The increased access to cellular targets afforded by EDTA would also be predicted to decrease the rates of resistance.

The use of antimicrobial lock solutions is becoming increasingly attractive to clinicians to extend the life span of CVADs and improve patient health (12). In critically ill patients or patients with long-term catheter use, if lines become blocked, there can sometimes be a lack of other suitable sites of entry (7). The antimicrobial killing effects shown in this study indicate that tetrasodium EDTA used as a catheter lock solution would reduce the chance of bacteria getting flushed into the body. In addition, repeated exposure of microorganisms to such a lock solution would be predicted to have a cumulative effect. Exposure times may often be increased from what we have tested here (i.e, hemodialysis patients for 24 to 72 h [17], oncology patients for 24 h to 3 weeks [8], and total parenteral nutrition patients for 12 to 24 h [66]).

Based on 2011 data from the World Health Organization and the U.S. Centers for Disease Control, the overall rates of infections acquired in hospital are 4.5% in the United States, 7.1% in Europe, and 11.6% in Canada (1). Published data indicate that each year in Canada, there are 50,000 catheter-associated bloodstream infections with approximately 12,500 related deaths and an estimated $1.2 billion dollars of health care expenditure to treat these infections. The use of a nonantibiotic, antimicrobial lock solution, like 4% tetrasodium EDTA, for preventative maintenance of catheters in patients fits well with antibiotic stewardship programs currently mandated around the world. This treatment would help to reduce the rates of CLABSI and other complications that are associated with long-term CVAD use.

## MATERIALS AND METHODS

### Isolation and identification of microorganisms from patient samples.

During an 8-month period at the Southlake Regional Health Centre in Newmarket, Ontario, 305 CVADs were collected from patients >18 years old. A 1- ml sample of the lock fluid was taken, CVADs were aseptically removed from patients, and the last distal 10 cm of the catheter was placed into a sterile collection tube. Sample collection and processing were done in compliance with protocols approved by the SRHC’s Research Ethics Board.

Catheter tip and lock solution samples were processed for microbiological culture in the laboratory of Tony Mazzulli (Mt. Sinai Hospital, Department of Microbiology, Toronto, ON). Following the methodology of Guembe and colleagues (34), each tip was rolled on a blood agar plate to detect extraluminal microorganisms (i.e., the Maki roll technique) and then cut into pieces into 5-ml brain heart infusion (BHI) medium followed by sonication to dislodge intraluminal microorganisms. After 1 min of sonication, a 0.1-ml aliquot of solution was removed and inoculated onto blood agar. The BHI/catheter tip solution was sonicated for an additional 4 min, and a 0.1-ml aliquot was removed and inoculated onto blood agar. For the lock solutions, the entire 1-ml sample was inoculated onto two blood agar plates. All blood agar plates were incubated at 35°C in 5% CO_2_ atmosphere for 24 or 48 h, and the total number of colonies of each type of isolate was recorded. For species identification, isolates were streak purified on blood agar plates and identified using the Vitek-MS automated mass spectrometry microbial identification system (bioMérieux). Susceptibility testing was performed following standard procedures with breakpoints recommended by the Clinical and Laboratory Standards Institute (CLSI). Glycerol stocks of each isolate were sent to VIDO-InterVac (Saskatoon, Saskatchewan [SK]) and used for all subsequent experiments.

Saskatchewan isolates were collected through the clinical microbiology laboratory at Royal University Hospital (Saskatoon, SK) from patient specimens that were submitted for culture and susceptibility testing. Organism identification was by matrix-assisted laser desorption ionization–time of flight mass spectrometry (MALDI-TOF). Initial antimicrobial susceptibility testing was performed with a Vitek II instrument using the GPS 67 card. For MRSA, organisms screening as resistant to oxacillin and cefoxitin were further tested for altered penicillin-binding protein (PBP) production using a latex agglutination assay. Confirmation using a PCR assay for the *mecA* gene was used if the susceptibility results and PBP assay did not agree. VRE isolates were confirmed for vancomycin resistance using Etest strips (bioMérieux). From blood agar, all isolates were inoculated into brain heart infusion (BHI) broth and grown for 18 h at 37°C, and freezer stocks were prepared in 20% glycerol.

### Conditions for routine growth of microorganisms.

Gram-positive and Gram-negative bacterial isolates were cultivated in Mueller-Hinton (MH) or Mueller-Hinton II (MH II) broth (Becton, Dickinson). Candida albicans and Candida glabrata isolates were grown in 1% tryptone broth supplemented with 0.5% glucose. Prior to each MIC, MBC, and MBEC experiment, isolates were inoculated from frozen stocks onto MH II agar and incubated at 37°C for 16 to 40 h. Individual colonies were used to inoculate 5 ml of the appropriate medium, and the culture was incubated at 37°C for 16 to 18 h with shaking at 200 rpm. These cultures were diluted to the desired cell concentrations and used for inoculation into 96-well plates.

### Determining the relationship between optical density and cell number for each microorganism.

To determine the conversion factor for cell number as a function of optical density for each strain, 1-ml stock cultures were prepared from overnight cultures to an optical density at 600 nm (OD_600_) of 1.0, and the cell number was determined by serial dilution and plating. The “drop dilution assay” consists of a 10-fold dilution series prepared in duplicate in a 96-well plate and 4-µl drops of the 10^−1^ to 10^−6^ dilutions inoculated in duplicate onto agar plates. After incubation at 37°C for 16 to 18 h, colonies were counted and recorded at the appropriate dilution that yielded between 3 and 30 colonies.

### Biofilm screening of microbial isolates.

Bacterial and fungal broth cultures were diluted to deliver 10^7^ cells (Gram-positive and Gram-negative species) or 10^5^ cells (Candida species) into wells of non-tissue-culture-treated, polystyrene 96-well plates (Falcon no. 351172). Growth was tested in a variety of media (Table S1) at 150 μl per well at 28 or 37°C for 24 or 48 h; inoculated plates were covered with lids, sealed with Parafilm, and incubated with slight rocking on a tilting platform shaker. Each isolate was tested with 6 to 8 replicates. Biofilm cell mass was quantitated by crystal violet (CV) staining (67). The 96-well plates were washed twice by being submerged into a water tray, followed by being shaken into a waste tray to remove nonattached cells. After air drying for 10 min, 125 µl of 0.1% (wt/vol) crystal violet solution was added to each well, and the plate was incubated for 10 to 15 min at room temperature. After staining, plates were washed twice with water and vigorously tapped on paper towels to remove any excess liquid, followed by air drying for 5 to 10 min. Two hundred microliters of 95% ethanol was added to each well, and the plates were covered and incubated for 15 min at room temperature. One hundred twenty-five microliters of solution from each well was transferred into a new clear flat-bottom 96-well plate (Greiner Bio One, no. 655101), and the optical density at 590 nm was measured. The best biofilm-forming isolates, as determined by CV staining, were further tested to determine the optimal conditions to facilitate maximum biofilm formation (Table 3). For Candida species, the biofilm growth conditions outlined by Serrano-Fujarte et al. (68) were used as a starting point.

**TABLE 3:** Optimal *in vitro* growth conditions for biofilm formation of microorganisms isolated from two Canadian hospitals

Organism type^*a*^	Biofilm medium^*b*^	Incubation conditions^*c*^
Gram-positive bacteria		
S. epidermidis		
ON	MH II + 2% NaCl	48 h at 37°C
SK	M9 + 0.5% glucose, 0.5% CAA	24 h at 37°C
S. aureus		
ON	TSB + 1.5% glucose	24 h at 37°C
Methicillin resistant		
ON	BHI + 2% glucose, 4% NaCl	48 h at 37°C
SK	BHI + 1% glucose, 4% NaCl	24 h at 37°C
E. faecalis		
ON	TSB/BHI + 1% glucose, 4% NaCl	48 h at 37°C
Vancomycin resistant (SK)	TSB + 2% glucose, 4% NaCl	48 h at 37°C
Gram-negative bacteria		
Escherichia coli		
ON	M9 + 0.2% glucose, 0.2% CAA	48 h at 28°C
SK	M63 + 0.3% glucose, 0.5% CAA	48 h at 28°C
Stenotrophomonas maltophilia (ON)	TSB + 0.5% glucose	48 h at 37°C
Pseudomonas aeruginosa (SK)	LB	24 h at 37°C
Enterobacter agglomerans (ON)	M9 + 0.1% glucose, 1.0% CAA	48 h at 28°C
Serratia marcescens (SK)	TSB	48 h at 28°C
Proteus mirabilis (ON)	M63 + 0.25% glucose, 0.5% CAA	24 h at 37°C
Klebsiella pneumoniae (SK)	M9 + 0.25% glucose, 0.5% CAA	24 h at 37°C
Fungi		
Candida albicans (ON or SK)	YPD/2% glucose	24 h at 37°C
Candida glabrata (ON or SK)	YNB	48 h at 28°C
Control bacteria		
Salmonella enterica serovar Typhimurium	1/2 LB no salt + 40 μM 2,2-dipyridyl	24 h at 37°C

a Microorganisms were obtained from the Southlake Regional Health Centre in Ontario (ON) or the Royal University Hospital in Saskatchewan (SK).

b MH II, Mueller-Hinton II broth; M9, M9 minimal medium; CAA, Casamino Acids; TSB, tryptic soy broth; BHI, brain heart infusion; M63, M63 minimal medium; LB, lysogeny broth; YPD/2% glucose, 1% yeast extract and 2% peptone supplemented with 2% glucose; YNB, yeast nitrogen base.

c For bacterial isolates, slight rocking was applied during growth; for fungal isolates, orbital shaking at 200 rpm was applied, which improved overall biofilm formation.

### Tetrasodium EDTA solution.

KiteLock 4% sterile catheter lock solution was supplied in 3-ml polypropylene ampoules (SterileCare, Inc.; Markham, ON, Canada). The patented solution is maintained at a high pH and contains 40 mg ml^−1^ of tetrasodium ethylenediamine tetraacetic acid (EDTA). We confirmed that a high pH was maintained for the tested range of dilutions used in all MIC, MBC, and MBEC assays (see Fig. S2 in the supplemental material).

10.1128/mSphere.00525-18.1 FIG S2 pH stability of the tetrasodium EDTA solution. The 4% solution was diluted in distilled water to the working concentrations used in MIC, MBC, and MBEC assays, and the pH was measured. Download FIG S2, TIF file, 0.2 MB. Copyright © 2018 Liu et al. 2018 Liu et al. This content is distributed under the terms of the Creative Commons Attribution 4.0 International license.

### MIC and MBC assays with tetrasodium EDTA.

MIC assays were performed using the broth microdilution method in 96-well plates, as recommended by the Clinical and Laboratory Standards Institute (CLSI [69]). In each assay, three biological replicates of one selected isolate were tested with 2 to 3 technical replicates. Tetrasodium EDTA was serially diluted 2-fold in the wells so that the final concentration ranged from 2% to 0.004%, and each well contained 50% growth medium, in a final volume of 100 μl. Wells were inoculated with 10^5^ bacterial cells or 2 × 10^3^ fungal cells, which are levels recommended by CLSI. Inoculated and uninoculated wells containing 100% growth medium were included as controls in each assay. The inoculated 96-well plates were covered with lids, sealed with Parafilm, and incubated at 37°C for 24 h with slight rocking on tilting platform shaker. The optical density of the cultures in each well was measured at 600 nm using a Victor X3 multilabel plate reader (Perkin Elmer). The MIC value was identified as the concentration breakpoint at which culture OD_600_ values were similar to those of uninoculated control wells. The MBC value was determined from the MIC plates by enumerating the viable bacterial cells in each well by drop dilution assay. MBC values were determined as the concentration resulting in CFU/ml values at the limit of detection (LOD [62.5 CFU ml^−1^]).

### MBEC assays with tetrasodium EDTA.

MBEC biofilm inoculator plates (Innovotech, AB, Canada), consisting of a 96-well plate bottom and a plastic lid with 96 polystyrene pegs attached, were used to grow biofilms, following procedures outlined by Harrison et al. (39). A total of 1.5 × 10^5^ (bacteria), 5 × 10^5^ (C. albicans), or 3 × 10^6^ (C. glabrata) cells were inoculated into wells containing the appropriate biofilm test medium (Table 3) to reach a final volume of 150 μl. For each isolate, two biological replicate cultures were tested in triplicate. The plates were sealed with Parafilm and incubated at 28 or 37°C for 24 or 48 h, with slight rocking for bacteria or orbital shaking at 200 rpm for fungal isolates. After biofilm growth, the pegs were washed twice in phosphate-buffered saline (PBS) for 2 min. Pegs in column 1 (*n* = 6) represented biofilm growth controls; after washing, these pegs were removed and analyzed as described below to determine the starting biofilm cell numbers.

For antimicrobial challenge, the pegs were placed into a new 96-well plate containing 200 μl of tetrasodium EDTA ranging from a final concentration of 4% to 0.008% in 50% growth medium. The last column of wells did not contain EDTA, and these pegs were used as the untreated control. The plate was sealed with Parafilm and incubated at 37°C for 24 h, with slight rocking on a platform shaker. After the challenge, the pegs attached to the lid were washed twice in PBS for 2 min and transferred to a 96-well plate containing 200 µl of recovery medium (growth medium supplemented with 1% Tween 80) in each well. The recovery plate was sealed with Parafilm, placed in a metal tray inside a Branson 3510 bath sonicator (Branson, Canada), and sonicated for 30 min. After sonication, drop dilution assays were performed to enumerate the viable cells dislodged from the pegs. MBEC values were determined as the concentration of tetrasodium EDTA that yielded a viable cell concentration at or near the LOD (125 CFU ml^−1^) for at least 50% of the biological and technical replicates. As a final test of killing, the lid with pegs was transferred into a plate containing 150 μl of growth medium per well, and cells were grown overnight. In each case where a value is reported at the LOD, there was no growth detected after 24 h.

### Control bacteria for MIC, MBC, and MBEC assays.

To ensure consistency and reproducibility from plate to plate for the MIC, MBC, and MBEC assays, two rows were inoculated with Salmonella enterica serovar Typhimurium ATCC 14028. In one row, tetrasodium EDTA was used at the same concentrations as the rest of the plate, and in the second row, gentamicin was used, ranging from a concentration of 400 μg ml^−1^ to 0.8 μg ml^−1^.

### Exposure time to eradicate biofilms (time-to-kill assays).

Bacterial and fungal biofilms were grown as described for the MBEC assays. After growth, control pegs (*n* = 16) were removed and analyzed to determine the starting biofilm cell numbers. After washing twice in PBS to remove growth medium, the biofilm pegs were exposed to 200 μl of 4% tetrasodium EDTA or sterile water (control) and incubated at 37°C for 1, 3, 6, or 24 h. Four biological replicates of each isolate were tested, with four technical replicates analyzed for 4% tetrasodium EDTA treatment and 2 technical replicates analyzed for water controls. After each exposure time, the biofilm cells were dislodged from the pegs by sonication, and viable cells were enumerated by drop dilution. Killing time was defined as the shortest exposure time that resulted in numbers of viable cells at or near the LOD (125 CFU ml^−1^) for at least 50% of the biological and technical replicates.

### Statistical analysis.

Statistical analysis of the data was performed using GraphPad Prism version 7.0c. For MBEC assays, the log reduction values (CFU/ml) at each test concentration were logarithmically transformed and evaluated using the Shapiro-Wilk normality test, which determined that the data were not normally distributed. A Kruskal-Wallis test was utilized to compare the differences between the log reductions at each test concentration and the appropriate medium control. For time-to-kill assays, unpaired *t* tests with Welch’s correction were used to compare CFU/ml values from 4% tetrasodium EDTA and water control treatments. For the Kruskal-Wallis and *t* tests, a *P* value of <0.05 was considered to be statistically significant.
