# Supplementary material for: Tetrasodium EDTA Is Effective at Eradicating Biofilms Formed by Clinically Relevant Microorganisms from Patients’ Central Venous Catheters
Source: mSphere. 2018 Nov 28;3(6):e00525-18. doi: 10.1128/mSphere.00525-18 (PMC6262258; doi:10.1128/mSphere.00525-18)
Supplement: TABLE S1 [file sph006182705st1.pdf]

Table S1. Biofilm screening conditions tested on SK and ON microbial isolates.

Bacterial species (No of isolates)[a]	Biofilm media tested[b]	Temperature Time	Reference[c]
Staphylococcus Epidermidis **(77)**	TSB<br>TSB + 0.5%, 1% or 2% glucose	37°C<br>24h or 48h	(1, 2)
	0.5 TSB + 1% glucose		(3)
	TSB + 1% glucose + 4% NaCl		
	MHII + 2% NaCl<br>BHI + 1% glucose + 4% NaCl,<br>M9 + 0.25% glucose + 0.5% CAA<br>M9 + 0.5% glucose + 1% CAA<br>M9 + 1.0% glucose + 2% CAA<br>M63 + 0.3% glucose + 0.5% CAA		(4)
Staphylococcus aureus **(10)**	TSB<br>TSB + 0.5%, 1%, 1.5%, or 2% glucose<br>TSB + 1% glucose + 4% NaCl	37°C<br>24h or 48h	(1)
	MHII + 2% NaCl<br>BHI + 0.5%, 1% or 2% glucose + 1% NaCl		(4)
	BHI + 1%, 2% or 3% glucose + 4% NaCl<br>M9 + 0.25%, 0.5%, or 1% glucose + 0.5%, 1% or 2% CAA<br>M63 + 0.3% glucose + 0.5% CAA		(5)
Enterococcus faecalis **(5)**	TSB<br>TSB + 0.25%, 0.5%, 1%, or 2% glucose<br>TSB + 1% glucose + 4% NaCl<br>MHII + 2% NaCl<br>BHI + 0.5%, 1% or 2% glucose + 1% NaCl<br>BHI + 1%, 2% or 3% glucose + 4% NaCl<br>M9 + 0.25%, 0.5% or 1% glucose + 0.5%, 1% or 2% CAA	37°C<br>24h or 48h	(6, 7)
	M9 + 0.5% yeast extract + 1% CAA<br>M63 + 0.3% glucose + 0.5% CAA		(8)
Escherichia coli **(5)**	TSB<br>TSB + 0.5%, 1%, or 2% glucose<br>TSB + 1% glucose + 4% NaCl<br>MHII + 2% NaCl<br>BHI + 1% glucose + 4% NaCl<br>M9 + 0.1%, 0.25%, 0.5% or 1% glucose + 0.2%, 0.5%, 1% or 2% CAA	37°C<br>24h or 48h	(9)
	M63 + 0.3% glucose + 0.5% CAA<br>LB		(10)

Bacterial species (No of isolates)[A]	Biofilm media tested[B]	Temperature Time	Reference[C]
***Stenotrophomonas maltophilia* (3)**	TSB	37°C	(11)
***Pseudomonas aeruginosa* (3)**	TSB + 0.5% glucose	24h or 48h	
	M9 + 0.25% glucose + 0.5% CAA		
	M63 + 0.3% glucose + 0.5% CAA		(12)
	LB		
***Enterobacter agglomerans* (4)**	BHI + 0.5%, 1% or 2% glucose + 1% NaCl	37°C	
	BHI + 1%, 2% or 3% glucose + 4% NaCl	24h or 48h	
	M9 + 0.1% or 0.2% glucose		
	M9 + 0.1% glucose + 0.5% or 1% CAA		
	M9 + 0.2% glucose + 0.2% CAA		
	M63 + 0.3% glucose + 0.5% CAA		
	LB		
***Serratia marcescens* (3)**	TSB	37°C	
	M9 + 0.25% glucose + 0.5% CAA	24h or 48h	
	M9 + 0.5% glucose		(13)
	LB		(14)
***Proteus mirabilis* (3)**	TSB	37°C	
	TSB + 0.5% glucose	24h or 48h	
	M63 + 0.3% glucose + 0.5% CAA		
	M9 + 0.25% glucose + 0.5% CAA		
	LB		
***Klebsiella pneumonia* (3)**	TSB	37°C	
	1/2 TSB	24h or 48h	
	M9 + 0.25% glucose + 0.5% CAA		(15)
	LB		(16)
***Candida albicans* (22)**	M9 + 0.25% glucose + 0.5% CAA	28ºC or 37°C	
***Candida glabrata* (7)**	RPMI 1640 + MOPS pH7	24h or 48h	(17)
	TSB		
	YPD + 2% glucose		(18)
	YNB + 0.9% glucose		(18)
***Salmonella* serovar Typhimurium (1)**	1/2 LB (no salt) + 40uM 2,2'-dipyridyl	28ºC or 37ºC 24h or 48h	(19)

[a] Each species is listed with the number of isolates screened in parentheses
[b] Abbreviations: TSB, Tryptic soy broth; MHII, Mueller Hinton II Broth; M9, M9 minimal media; CAA, Casamino acids; M63, M63 minimal media; BHI, Brain Heart Infusion; LB, Lysogeny broth; MOPS, 3-(*N*-morpholino)propanesulfonic acid; YPD, yeast extract-peptone-dextrose; YNB, yeast nitrogen base
[c] References were used as the starting point for choosing growth media. For consistency of testing, each strain was grown in TSB.

REFERENCES

1. **Christensen GD**, **Simpson WA**, **Younger JJ**, **Baddour LM**, **Barrett FF**, **Melton DM**, **Beachey EH**. 1985. Adherence of coagulase-negative staphylococci to plastic tissue culture plates: a quantitative model for the adherence of staphylococci to medical devices. Journal of Clinical Microbiology **22**:996–1006.

2. **Ziebuhr W**, **Heilmann C**, **Götz F**, **Meyer P**, **Wilms K**, **Straube E**, **Hacker J**. 1997. Detection of the intercellular adhesion gene cluster (ica) and phase variation in Staphylococcus epidermidis blood culture strains and mucosal isolates. Infection and Immunity **65**:890–896.

3. **Opperman TJ**, **Kwasny SM**, **Williams JD**, **Khan AR**, **Peet NP**, **Moir DT**, **Bowlin TL**. 2009. Aryl rhodanines specifically inhibit staphylococcal and enterococcal biofilm formation. Antimicrobial Agents and Chemotherapy **53**:4357–4367.

4. **Saginur R**, **Stdenis M**, **Ferris W**, **Aaron SD**, **Chan F**, **Lee C**, **Ramotar K**. 2006. Multiple combination bactericidal testing of staphylococcal biofilms from implant-associated infections. Antimicrobial Agents and Chemotherapy **50**:55–61.

5. **O'Neill E**, **Pozzi C**, **Houston P**, **Humphreys H**, **Robinson DA**, **Loughman A**, **Foster TJ**, **O'Gara JP**. 2008. A novel Staphylococcus aureus biofilm phenotype mediated by the fibronectin-binding proteins, FnBPA and FnBPB. Journal of Bacteriology **190**:3835–3850.

6. **Toledo-Arana A**, **Valle J**, **Solano C**, **Arrizubieta MJ**, **Cucarella C**, **Lamata M**, **Amorena B**, **Leiva J**, **Penadés JR**, **Lasa I**. 2001. The enterococcal surface protein, Esp, is involved in Enterococcus faecalis biofilm formation. Applied and Environmental Microbiology **67**:4538–4545.

7. **Holmberg A**, **Mörgelin M**, **Rasmussen M**. 2012. Effectiveness of ciprofloxacin or linezolid in combination with rifampicin against Enterococcus faecalis in biofilms. J Antimicrob Chemother **67**:433–439.

8. **Dunny GM**, **Clewell DB**. 1975. Transmissible toxin (hemolysin) plasmid in Streptococcus faecalis and its mobilization of a noninfectious drug resistance plasmid. Journal of Bacteriology **124**:784–790.

9. **Carneiro VA**, **Santos HSD**, **Arruda FVS**, **Bandeira PN**, **Albuquerque MRJR**, **Pereira MO**, **Henriques M**, **Cavada BS**, **Teixeira EH**. 2010. Casbane diterpene as a promising natural antimicrobial agent against biofilm-associated infections. Molecules **16**:190–201.

10. **Chassaing B**, **Darfeuille-Michaud A**. 2013. Adherent-invasive Escherichia coli Biofilm Formation Assays. BIO-PROTOCOL **3: e982**.

11. **Peeters E**, **Nelis HJ**, **Coenye T**. 2008. Comparison of multiple methods for quantification of microbial biofilms grown in microtiter plates. J Microbiol Methods **72**:157–165.

12. **Drenkard E**, **Ausubel FM**. 2002. Pseudomonas biofilm formation and antibiotic resistance are linked to phenotypic variation. Nature **416**:740–743.

13. **Koh KS**, **Lam KW**, **Alhede M**, **Queck SY**, **Labbate M**, **Kjelleberg S**, **Rice SA**. 2007. Phenotypic diversification and adaptation of Serratia marcescens MG1 biofilm-derived morphotypes. Journal of Bacteriology **189**:119–130.

14. **Shanks RMQ**, **Stella NA**, **Kalivoda EJ**, **Doe MR**, **O'Dee DM**, **Lathrop KL**, **Guo FL**, **Nau GJ**. 2007. A Serratia marcescens OxyR Homolog Mediates Surface Attachment and Biofilm Formation. Journal of Bacteriology **189**:7262–7272.

15. **Chhibber S**, **Nag D**, **Bansal S**. 2013. Inhibiting biofilm formation by Klebsiella pneumoniae B5055 using an iron antagonizing molecule and a bacteriophage. BMC Microbiol **13**:174.

16. **Maldonado NC**, **de Ruiz CS**, **Cecilia M**, **Nader-Macias ME**. 2007. A simple technique to detect Klebsiella biofilm-forming-strains. Inhibitory potential of Lactobacillus fermentum CRL 1058 whole cells and products. Communicating Current Research and Educational Topics and Trends in Applied Microbiology 52–58.

17. **Melo AS**, **Bizerra FC**, **Freymüller E**, **Arthington-Skaggs BA**, **Colombo AL**. 2011. Biofilm production and evaluation of antifungal susceptibility amongst clinical Candida spp. isolates, including strains of the Candida parapsilosis complex. Medical Mycology, **49**:253–262.

18. **Serrano-Fujarte I**, **López-Romero E**, **Reyna-López GE**, **Martínez-Gámez MA**, **Vega-González A**, **Cuéllar-Cruz M**. 2015. Influence of Culture Media on Biofilm Formation by CandidaSpecies and Response of Sessile Cells to Antifungals and Oxidative Stress. BioMed Research International **2015**:1–15.

19. **White AP**, **Gibson DL**, **Grassl GA**, **Kay WW**, **Finlay BB**, **Vallance BA**, **Surette MG**. 2008. Aggregation via the Red, Dry, and Rough Morphotype Is Not a Virulence Adaptation in Salmonella enterica Serovar Typhimurium. Infection and Immunity **76**:1048–1058.
