# Supplementary material for: Tetrasodium EDTA Is Effective at Eradicating Biofilms Formed by Clinically Relevant Microorganisms from Patients’ Central Venous Catheters
Source: mSphere. 2018 Nov 28;3(6):e00525-18. doi: 10.1128/mSphere.00525-18 (PMC6262258; doi:10.1128/mSphere.00525-18)
Supplement: TABLE S2 [file sph006182705st2.pdf]

Table S2. Antibiotic susceptibility profiles for biofilm-forming microbes isolated from Ontario and Saskatchewan hospitals.

Microbial isolates[A]	Antimicrobial Agents[B]																	
	FOX	PEN	AMP	OXA	GEN	STR	CIP	LVX	MXF	ERY	CLI	LZD	VAN	TET	TGC	NIT	RIF	SXT
**Gram-positive bacteria**																		
S. epidermidis (ON)	POS	>=0.5 (R)[C]		>=4 (R)			>=8 (R)		>=8 (R)	<=0.25 (S)	<=0.25 (S)	2 (S)	<=0.5 (S)	2 (S)	<=0.12 (S)	<=16 (S)	<=0.5 (S)	160 (R)
S. epidermidis (SK)	NEG	<=0.03 (S)		<=0.25 (S)			<=0.5 (S)		<=0.25 (S)	>=8 (R)	<=0.25 (S)	1 (S)	<=0.5 (S)	<=1 (S)	<=0.12 (S)	<=16 (S)	<=0.5 (S)	<=10 (S)
S. aureus (ON)	NEG	<=0.03 (S)		<=0.25 (S)			<=0.5 (S)		<=0.25 (S)	<=0.25 (S)	<=0.25 (S)	2 (S)	<=0.5 (S)	<=1 (S)	<=0.12 (S)	<=16 (S)	<=0.5 (S)	<=10 (S)
Methicillin-resistant *S. aureus* (ON)	POS	>=0.5 (R)		>=4 (R)			>=8 (R)		<=0.25 (S)	>=8 (R)	>=8 (R)	2 (S)	<=0.5 (S)	<=1 (S)	<=0.12 (S)	<=16 (S)	<=0.5 (S)	<=10 (S)
Methicillin-resistant *S. aureus* (SK)	POS	>=0.5 (R)		>=4 (R)			<=0.5 (S)		<=0.25 (S)	<=0.25 (S)	<=0.25 (S)	2 (S)	<=0.5 (S)	<=1 (S)	<=0.12 (S)	<=16 (S)	<=0.5 (S)	<=10 (S)
E. faecalis (ON)		1 (R)	<=2 (S)	>=4 (R)	SYN-S (S)	SYN-S (S)	<=0.5 (S)	0.5 (S)		1 (R)	<=0.25 (S)	2 (S)	1 (S)	<=1 (S)	<=0.12 (S)	<=16 (S)		
Vancomycin-resistant *E. faecalis* (SK)		>= 64 (R)	>= 32 (R)		SYN-S (S)	SYN-S (S)	>=8 (R)	>=8 (R)		>=8 (R)		2 (S)	>=32 (R)	>=16 (R)	<=0.12 (S)	128 (R)		

	AMP	AMC	TZP	CFZ	FOX	CPD	CTX	CAZ	CRO	ETP	MEM	AMK	GEN	TOB	CIP	TGC	NIT	SXT
**Gram-negative bacteria**																		
Escherichia coli (ON)	8 (S)	4 (S)	<=4 (S)	<=4 (S)	<=4 (S)	0.5 (S)	<=1 (S)	<=1 (S)	<=1 (S)	<=0.5 (S)	<=0.25 (S)	<=2 (S)	<=1 (S)	<=1 (S)	<=0.25 (S)	<=0.5 (S)	<=16 (S)	<=20 (S)
Escherichia coli (SK)	<=2 (S)	<=2 (S)	<=4 (S)	<=4 (S)	<=4 (S)	<=0.25 (S)	<=1 (S)	<=1 (S)	<=1 (S)	<=0.5 (S)	<=0.25 (S)	<=2 (S)	<=1 (S)	<=1 (S)	<=0.25 (S)	<=0.5 (S)	<=16 (S)	<=20 (S)
Stenotrophomonas maltophilia (ON)																		<=20 (S)
Pseudomonas aeruginosa (SK)			8 (S)				32 (R)	4 (S)			<=0.25 (S)	<=2 (S)	<=1 (S)	<=1 (S)	<=0.25 (S)	>=8 (R)		
Enterobacter agglomerans (ON)																		
Serratia marcescens (SK)	16 (R)	>=32 (R)		>=64 (R)	16 (R)		<=1 (S)	<=1 (S)	<=1 (S)	<=0.5 (S)	<=0.25 (S)	<=2 (S)	<=1 (S)	8 (R)	<=0.25 (S)	1 (S)	256 (R)	<=20 (S)
Proteus mirabilis (ON)	<=2 (S)	<=2 (S)	<=4 (S)	<=4 (S)	<=4 (S)		<=1 (S)	<=1 (S)	<=1 (S)	<=0.5 (S)	<=0.25 (S)	<=2 (S)	<=1 (S)	<=1 (S)	<=0.25 (S)	4 (R)	128 (R)	>=320 (R)
Klebsiella pneumoniae (SK)	>=32 (R)	4 (S)	16 (S)	<=4 (S)	>=64 (R)	2 (S)	<=1 (S)	<=1 (S)	<=1 (S)	<=0.5 (S)	<=0.25 (S)	<=2 (S)	<=1 (S)	<=1 (S)	>=4 (R)	>=8 (R)	128 (R)	>=320 (R)
**Control bacteria**																		
Salmonella serovar Typhimurium	<=2 (S)	<=2 (S)	<=4 (S)	<=4 (S)	<=4 (S)	<=0.25 (S)	<=1 (S)	<=1 (S)	<=1 (S)	<=0.5 (S)	<=0.25 (S)	<=2 (S)	<=1 (S)	<=1 (S)		<=0.5 (S)	32 (S)	<=20 (S)

	FLC	VRC	CAS	MFG	AMB	5FC
**Fungi**						
Candida albicans (ON)	<=0.5 (S)	<=0.12 (S)	<=0.12 (S)	<=0.06 (S)	2 (R)	<=1 (S)
Candida albicans (SK)	<=0.5 (S)	<=0.12 (S)	<=0.12 (S)	<=0.06 (S)	<=0.25 (S)	<=1 (S)
Candida glabrata (ON)			<=0.12 (S)	<=0.06 (S)	<=0.25 (S)	<=1 (S)
Candida glabrata (SK)			<=0.12 (S)	<=0.06 (S)	<=0.25 (S)	<=1 (S)

[A]Isolates were inoculated from freezer stocks and propagated on TSA-blood agar or Sabouraud-blood agar at 37ºC, prior to analysis on the Vitek II system (Biomerieux). Gram-positive isolates were analyzed using the AST-GP67 card; Gram-negative isolates were analyzed using the AST-N219 card; Fungal isolates were analyzed using the AST-YS08 card.

[B] Abbreviations: FOX-cefoxitin; PEN-benzylpenicillin; AMP-ampicillin; OXA-oxacillin; GEN-gentamycin; STR-streptomycin; CIP-ciprofloxacin; LVX-levofloxacin; MXF-moxifloxacin; ERY-erythromycin; CLI-clindamycin; LZD-linezolid; VAN-vancomycin; TET-tetracycline; TGC-tigecycline; NIT-nitrofurantoin; RIF-rifampicin; SXT-trimethoprim/sulfamethoxazole; AMC-amoxicillin/clavulanic acid; TZP-piperacillin/tazobactam; CFZ-cefazolin; FOX-cefoxitin; CPD-cefpodoxime; CTX-cefotaxime; CAZ-cefotaxime; CAZ-ceftazidime; CRO-ceftriaxone; ETP-ertapenem; MEM-meropenem; AMK-amikacin; TOB-tobramycin; FLC-fluconazole; VRC-voriconazole; CAS-caspofungin; MFG-micafungin; AMB-amphotericin B; 5FC-flucytosine

[C] MIC values are shown and isolates were designated as susceptible (S) or resitant (R) based on CLSI guidelines.
