# Supplementary figures and images for: Tetrasodium EDTA Is Effective at Eradicating Biofilms Formed by Clinically Relevant Microorganisms from Patients’ Central Venous Catheters
Source: mSphere. 2018 Nov 28;3(6):e00525-18. doi: 10.1128/mSphere.00525-18 (PMC6262258; doi:10.1128/mSphere.00525-18)

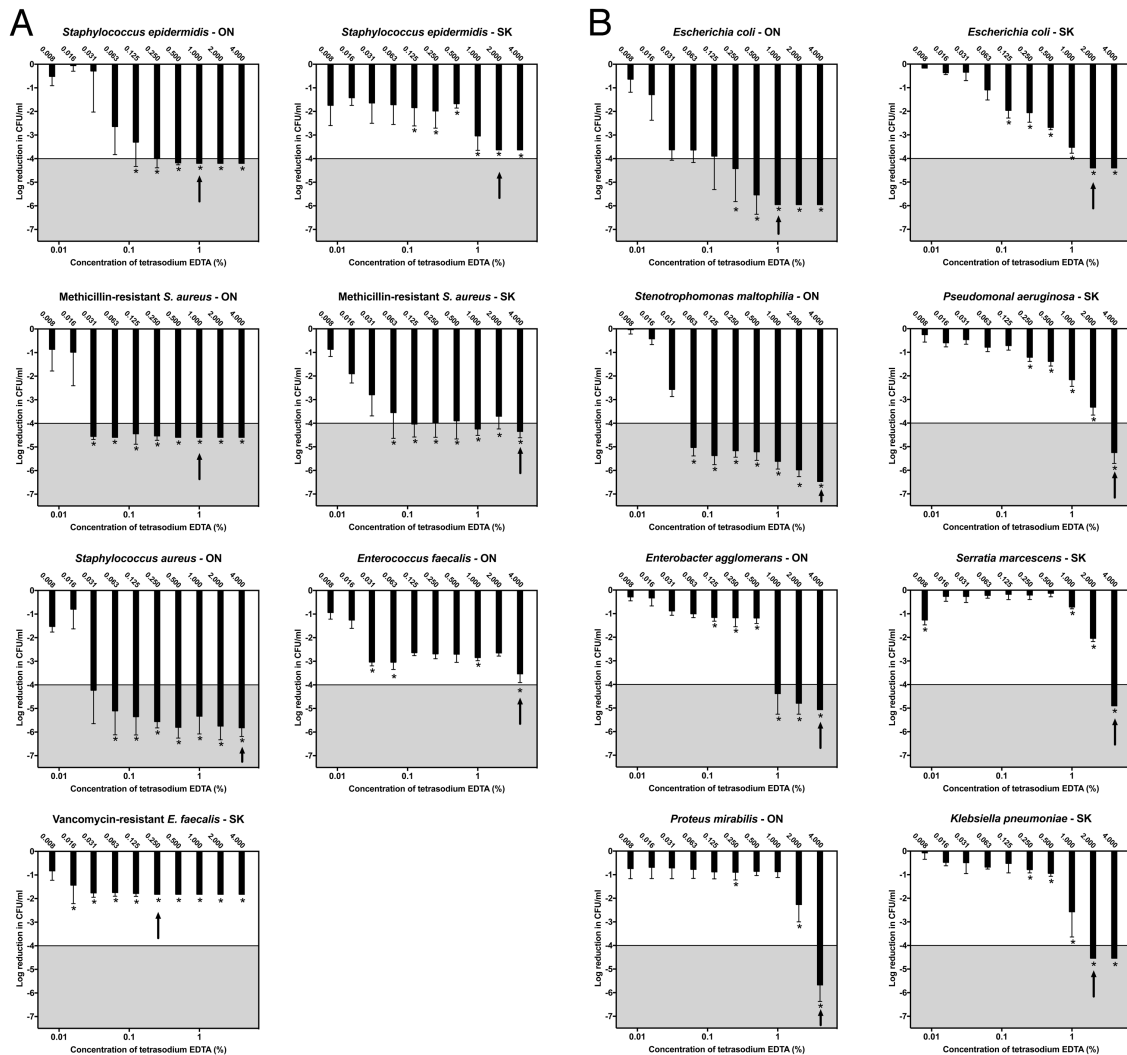

C

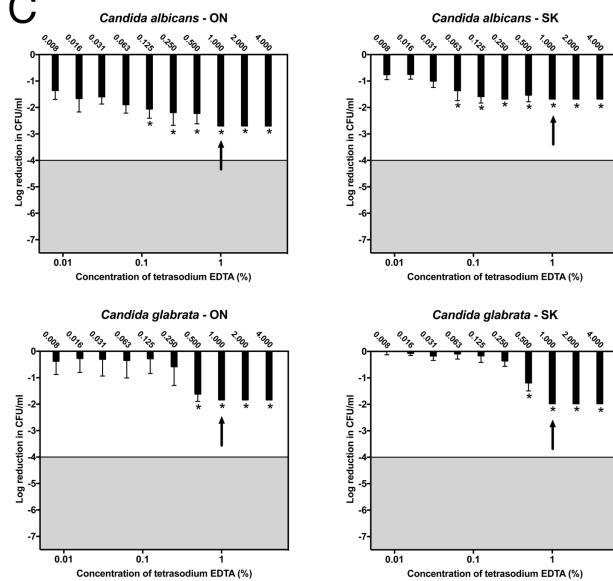

D

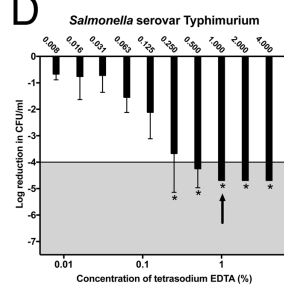

Supplement: FIG S1 [file sph006182705sf1.pdf]

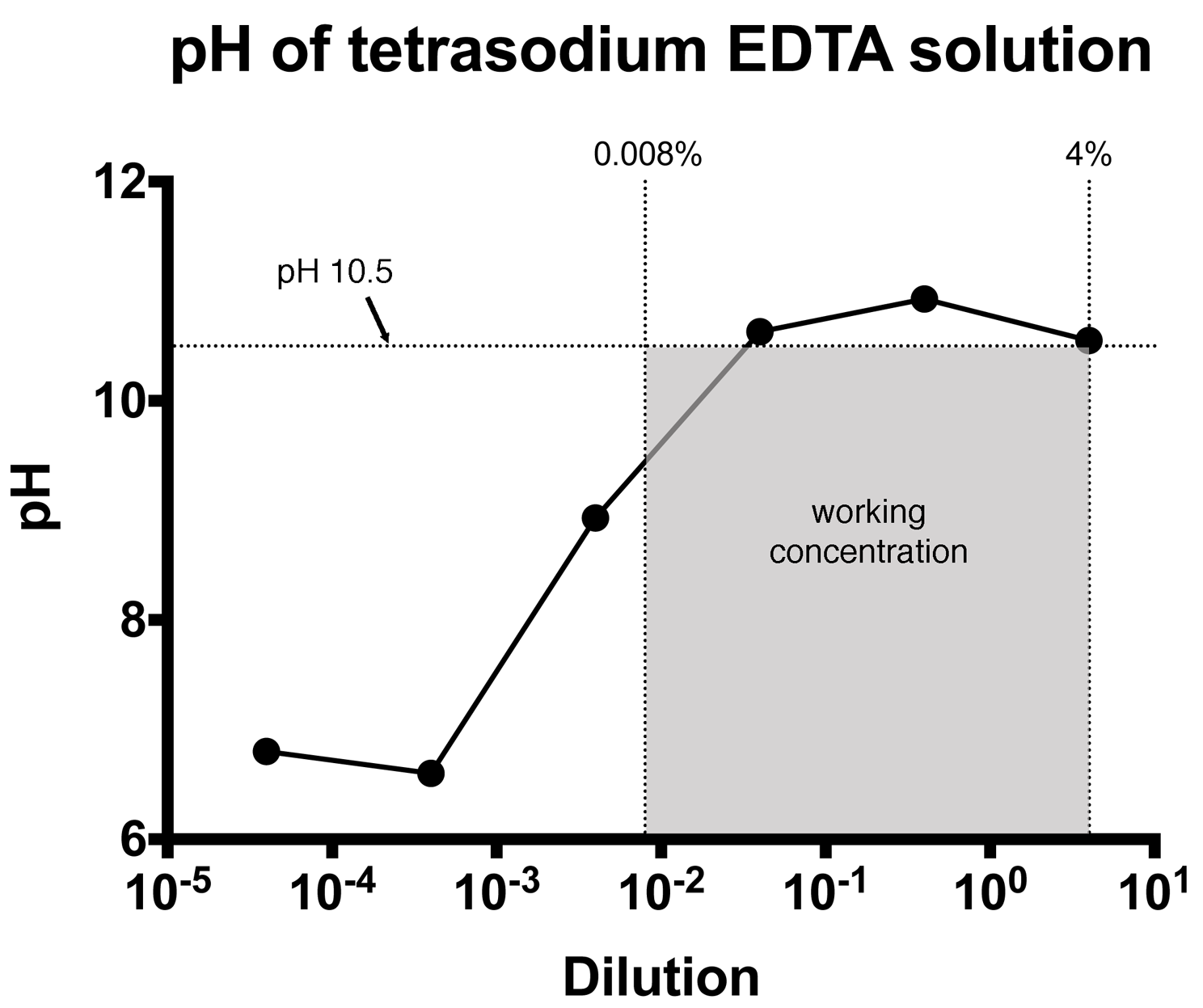

Supplement: FIG S2 [file sph006182705sf2.tif]
